# Detecting the Interdisciplinary Nature and Topic Hotspots of Robotics in Surgery: Social Network Analysis and Bibliometric Study

**DOI:** 10.2196/12625

**Published:** 2019-03-26

**Authors:** Lining Shen, Shimin Wang, Wei Dai, Zhiguo Zhang

**Affiliations:** 1 School of Medicine and Health Management Tongji Medical College Huazhong University of Science & Technology Wuhan China; 2 Institute of Smart Health Huazhong University of Science & Technology Wuhan China; 3 Hubei Provincial Research Center for Health Technology Assessment Wuhan China

**Keywords:** robotics, robotic surgery, interdisciplinary collaboration, topic hotspot, topic bursts, co-word analysis, bibliometric analysis, bibliometrics, social network analysis, robotic surgical procedures, laparoscopy

## Abstract

**Background:**

With the widespread application of a robot to surgery, growing literature related to robotics in surgery (RS) documents widespread concerns from scientific researchers worldwide. Although such application is helpful to considerably improve the accuracy of surgery, we still lack the understanding of the multidiscipline-crossing status and topic distribution related to RS.

**Objective:**

The aim of this study was to detect the interdisciplinary nature and topic hotspots on RS by analyzing the current publication outputs related to RS.

**Methods:**

The authors collected publications related to RS in the last 21 years, indexed by the Web of Science Core Collection. Various bibliometric methods and tools were used, including literature distribution analysis at the country and institution level and interdisciplinary collaboration analysis in the different periods of time. Co-word analysis was performed based on the keywords with high frequency. The temporal visualization bar presented the evolution of topics over time.

**Results:**

A total of 7732 bibliographic records related to RS were identified. The United States plays a leading role in the publication output related to RS, followed by Italy and Germany. It should be noted that the Yonsei University in South Korea published the highest number of RS-related publications. Furthermore, the interdisciplinary collaboration is uneven; the number of disciplines involved in each paper dropped from the initial 1.60 to the current 1.31. Surgery; Engineering; Radiology, Nuclear Medicine, and Medical Imaging; and Neurosciences and Neurology are the 4 core disciplines in the field of RS, all of which have extensive cooperation with other disciplines. The distribution of topic hotspots is in imbalanced status, which can be categorized into 7 clusters. Moreover, 3 areas about the evolution of topic were identified, namely (1) the exploration of techniques that make RS implemented, (2) rapid development of robotic systems and related applications in surgery, and (3) application of a robot to excision of tissues or organs targeted at various specific diseases.

**Conclusions:**

This study provided important insights into the interdisciplinary nature related to RS, which indicates that the researchers with different disciplinary backgrounds should strengthen cooperation to publish a high-quality output. The research topic hotspots related to RS are relatively scattered, which has begun to turn to the application of RS targeted at specific diseases. Our study is helpful to provide a potential guide to the direction of the field of RS for future research in the field of RS.

## Introduction

### Background

A robot is a mechatronic device that can be programmed to perform some tasks automatically, the emergence of which has significantly improved people’s quality of life. Apart from revolutionizing the manufacturing sector, robots have now found their way out of the factory and into applications such as agriculture, aerospace, and education [1], with no exception of medicine. One of the advantages of surgical robots over traditional surgery is that they are smart and precise and can accomplish their purpose more accurately. Dexterous robots have emerged in the last decade in response to the need for fine motor control assistance in applications as diverse as surgery, undersea welding, and mechanical manipulation in space [2]. For example, robots have also been integrated into operating rooms around the world and have enabled or improved many new minimally invasive surgical procedures [3-5].

Growing literature related to robotics in surgery (RS) documents widespread concerns from scientific researchers worldwide. As described in the literature, robots were introduced to the medical industry in the last century, initially for auxiliary work such as nursing and image transmission, so that doctors can get better examination results [6]. For example, installing a camera on the robotic arm and then sending the captured results to the screen can help the doctor to perform a more accurate examination for patients. Some researchers pointed out that surgical robots have also been used to try to solve some intractable diseases and increase the accuracy and safety of surgery [7,8]. Furthermore, with the development of information technology, the application of surgical robots has not only been limited to examination functions. However, other researchers believed that long-term feasibility still needs further assessment, although the treatment effect is acceptable in the short term [9]. In addition, the greatest surgical innovation of the past 3 decades has been the emergence of minimally invasive surgery in which many surgical robots are currently used. Therefore, the effect of minimally invasive surgery will also be greatly improved by means of a magnified view and improved ergonomics and dexterity provided by robotic platforms [6], which can reduce patient discomfort, costs, and hospital time [1]. However, limited data are available regarding safety and efficacy [10]. Therefore, some researchers compared the difference between robotic surgery and traditional surgery in detail [11] and evaluated the clinical effectiveness of surgical robots through function and outcomes [12].

Obviously, scientific researchers mainly focused on the clinical applications of surgical robots. However, to the best of our knowledge, little is known about the research situation, interdisciplinary nature, and research hotspots related to RS from the perspective of bibliometrics, which can help us comprehensively understand the process of the evolution of the related disciplines and research themes involved in RS.

### Objectives

This study analyzed RS-related publications from the perspective of bibliometrics to address the above limitations. Specifically, the purposes of this study were listed as follows:

1. How did the literature in the field of RS be distributed worldwide?
2. What was the interdisciplinary collaboration of RS in the last decades?
3. What were the topic hotspots and evolution process in the field of RS?

## Methods

### Sample and Data Collection

In this study, we chose publications indexed in the Web of Science Core Collection (WoSCC) database as the data source. As WoSCC adheres to a strict evaluation process and provides the most influential, relevant, and credible information, it is most suitable for subsequent bibliometric analysis in this study [13].

To fully retrieve RS-related publications, combining with the above literature review on RS, we constructed the following search strategy: #1 TS=((“robot* AND *assist*”) AND *surg*); #2 TS=((“robot* AND *guid*”) AND *surg*); #3 TI=(robot*AND *surg*); #4 #1 OR #2 OR #3. Moreover, the document type was limited to article and review; the time span of publications covered the period from 1986 to 2017.

Subsequently, a total of 10,087 bibliographic records were identified and downloaded on September 20, 2018. To perfect the research, the main inclusion and exclusion criteria were formulated after 2 researchers independently reviewed and evaluated the 1000 pilot bibliographic records. The inclusion criteria were as follows: (1) the content of the papers primarily focused on RS and (2) all study designs. The main exclusion criteria were as follows: (1) the record had no subject categorizations or keywords (eg, book review and notification), (2) the study merely mentioned robotic surgery as one of the surgical approaches [14], and (3) the content of the research did not focus on RS but patients with no robotic surgery [15]. Any discrepancies were discussed until consensus was reached in this process. Subsequently, 1 researcher reviewed the remaining records according to the above criteria. Finally, a total of 7732 bibliographic records were obtained for further bibliometric analysis, with 2355 inappropriate or irrelevant records removed, so as to elucidate the interdisciplinary nature and research topic hotspots in the field of RS internationally.

### Design of Data Analysis Method

There are various indicators used in the study to better demonstrate distribution of the literature. The total local citation score (TLCS) and the total global citation score (TGCS) were calculated in this study, which have been the key indicators capable of evaluating the relevance of each research paper in our sample [16]. TLCS refers to the number of times that a set of papers included in a collection has been cited by other papers within the same collection, whereas TGCS refers to the number of times that a set of papers included in a collection has been cited in the WoSCC [17]. In addition, the average global citation score (AGCS) is the mean value of TGCS. However, it should be noted that TLCS presents the important papers in a chosen research area, whereas TGCS mainly displays the effects of the papers related to a chosen research area on the papers in the WoSCC [18]. Distribution of the literature was presented using the HistCite tool, which is an analysis and visualization software that helps us to obtain information at the country and institution level [19]. Meanwhile, we divided the 21 years into 4 periods of time to exam the distribution at the country level.

Furthermore, interdisciplinarity and cross-disciplinarity have been buzzwords for the last few years, which are used to describe contributions from and collaborations among several or more disciplines. Interdisciplinary means that the content of research is not only a method or ability in a field but a field that involves more [20]. Through interdisciplinary research, we can more comprehensively understand the research content of a field. Interdisciplinary inevitably exists between disciplines, indicating that the scope involved in a certain field is constantly expanding [21]. Meanwhile, research areas constitute a subject categorization scheme that is shared by all Web of Science product databases. The literature indexed by WoSCC is assigned to at least 1 subject category, which is mapped to 1 research area [22]. VOSviewer—a software tool developed by Nees Jan van Eck and Ludo Waltman at Leiden University's Centre for Science and Technology Studies [23]—was employed to visualize the interdisciplinary collaboration on the basis of subject categorization of publication [24]. Each node represents a discipline, whereas the connection between nodes represents collaborations between disciplines. In addition, nodes with a close connection are assigned the same color to form their respective clusters. Furthermore, a co-occurrence matrix was generated by using the Bibliographic Item Co-occurrence Mining System (BICOMS) [25] to calculate the centrality, which includes degree centrality, closeness centrality, and betweenness centrality by using Ucinet6.6 [26]. Degree centrality is simply the number of tie of a given type that a node has; closeness is an inverse measure of centrality in the sense that large numbers indicate that a node is highly peripheral, whereas small numbers indicate that a node is more central; betweenness centrality is a measure of how often a given node falls along the shortest path between 2 other nodes [27]. Moreover, we analyzed the centrality in the different periods of time based on the top 5 centralities over the period from 1997 to 2017.

In addition, we used Cortext to visualize the evolution of individual disciplines and interdisciplinary clusters. The tubes layout represents the transformation of cluster of discipline over time [28-30]. The width of tubes represents the number of records in which they appear in the same cluster. Darker tubes mean more disciplines are shared between 2 consecutive time periods.

Finally, 3 stages were completed, as follows, regarding the analysis of research hotspots. First, BICOMS was employed to calculate the frequency of keywords. Subsequently, a total of 13,706 keywords were obtained and merged based on the following 4 criteria [31]: (1) merging some keywords into corresponding Medical Subject Headings terms using PubMed (eg, “gynaecology” and “lymphadenectomy” were merged into “gynecology” and “lymph node excision,” respectively); (2) unifying the uppercase and lowercase of some keywords (eg, “Laparoscopy” and “Bladder cancer” were changed to “laparoscopy” and “bladder cancer,” respectively); (3) standardizing the singular and plural of keywords (eg, “child” and “pediatric” were changed to “children” and “pediatrics,” respectively); and (4) merging some synonym keywords (eg, “minimal invasive surgery” and “MIS” were replaced by “minimally invasive surgery”). After merging, 90 keywords with frequencies not less than 40 were obtained.

Second, we used BICOMS to generate the 88×88 co-occurrence matrix of keywords with a frequency not less than 40. It is worth noting that we removed robotic surgery and surgical robot because they are our research object. Then, a social network map was drawn with respect to these 88 keywords by Ucinet6.6 and VOSviewer [26,32,33], which intuitively reflects the relationship between keywords of high frequency. The relative size of nodes is proportional to the frequency of keywords, whereas the relative width of lines is proportional to the correlation between keywords [34].

Third, we detected the burst strength of the cleaned keywords and drew a temporal bar graph for high-burst strength keywords. Burst strength depicts the intensity of the burst, that is, how great the change is in the word frequency that triggered the burst. Kleinberg burst detection algorithm [35] can recognize the sudden increase of word frequency over time and detect the burst of keyword popularity effectively. We chose Science of Science (Sci2) [36], which can implement this algorithm to find out the burst terms in the processed data and calculate the burst strength. Finally, 48 keywords with a burst strength of not less than 4 were obtained. However, these keywords may only be core keywords to a certain extent. Further screening by word frequency can improve the quality of core keywords. The higher the number of keyword frequency, the more likely it is to become a hot topic in future. Then we drew a temporal visualization map of 26 keywords with a frequency no less than 40 and burst strength more than 4 by Sci2 [37]. Each keyword has its own starting and ending time, and the area of each bar reflects its burst strength.

## Results

### Literature Distribution

**Figure 1:**
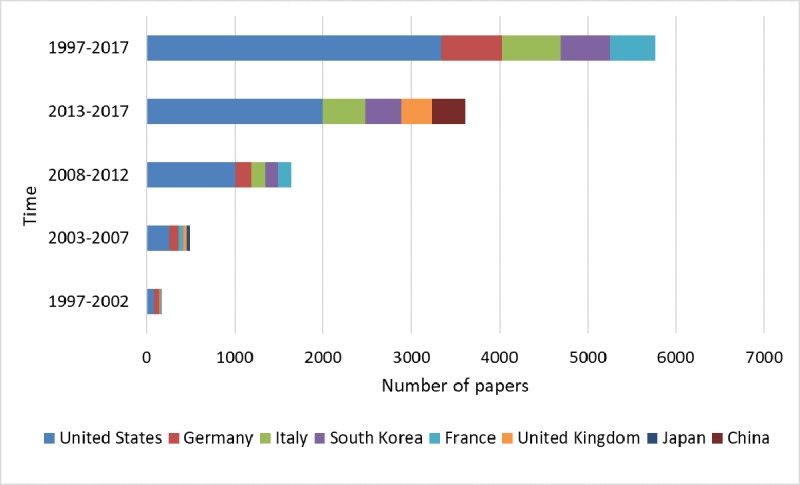
Top 5 countries of robotics in surgery-related research in each period of time.

A total of 22,470 authors were identified with 7732 papers, which are affiliated to 4721 institutions from 74 countries. All of these papers were published in 1030 journals with 105,835 citations. In total, 12 publication languages were included, of which, English ranks the first, followed by German and French. It can be found that the quantity of literature related to RS is growing rapidly over time, and the United States takes the top spot at every time slice (Figure 1). It should be noted that the developed countries had contributed to the majority of the publication, although China is among the top 10 (for the publication output of top 10 countries, see Multimedia Appendix 1). In addition, South Korea ranked the fifth in the publication output from 2008 to 2012 and 2013 to 2017. The distribution of institutions is shown in Table 1. Obviously, Yonsei University in South Korea takes the first place, with the highest TLCS and TGCS, followed by Cleveland Clinic and Mayo Clinic. Memorial Sloan-Kettering Cancer Center has the highest AGCS, with high academic influence and collaboration in RS-related research, followed by Stanford University.

**Table 1:** Distribution of the top 10 institutions with robotics in surgery-related research.

Number	Institution	Recs^a^	Publication, %	Cumulative percentage	TLCS^b^	TGCS^c^	AGCS^d^
1	Yonsei University	197	2.55	2.55	1498	3579	18.17
2	Cleveland Clinic	167	2.16	4.71	990	3268	19.57
3	Mayo Clinic	111	1.44	6.15	978	2674	24.09
4	The Johns Hopkins University	104	1.35	7.50	701	3485	33.51
5	University of Pittsburgh	102	1.32	8.82	416	2108	20.67
6	Imperial College London	98	1.27	10.09	424	1799	18.36
7	Harvard University	96	1.24	11.33	714	2759	28.74
8	Stanford University	90	1.16	12.49	908	3327	36.97
9	Vanderbilt University	83	1.07	13.56	585	1986	23.93
10	Memorial Sloan-Kettering Cancer Center	78	1.01	14.57	867	3262	41.82

^a^Recs: number of published papers.

^b^TLCS: total local citation score.

^c^TGCS: total global citation score.

^d^AGCS: average global citation score.

### Interdisciplinary Nature

#### Visualization of the Interdisciplinary Collaboration on Robotics in Surgery–Related Research

The number of disciplines is increasing over the 4 time slices; a total of 91 disciplines are involved as shown in Table 2. The disciplines covered by RS have expanded from 34 in the first 6 years to 85 in the last 5 years. A downward trend is presented for the average number of disciplines in each paper, from 1.60 to 1.31.

**Figure 2:**
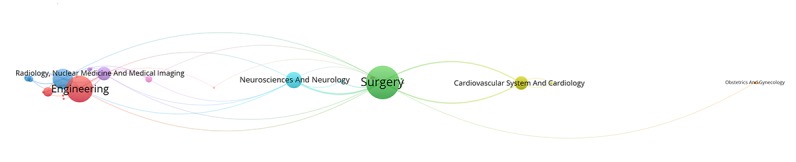
Interdisciplinary collaboration on robotics in surgery-related research from 1997 to 2002.

**Figure 3:**
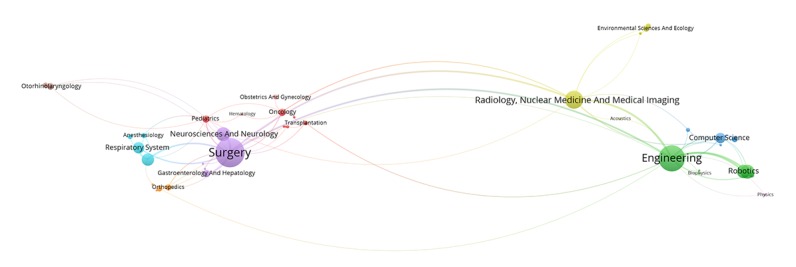
Interdisciplinary collaboration on robotics in surgery-related research from 2003 to 2007.

**Figure 4:**
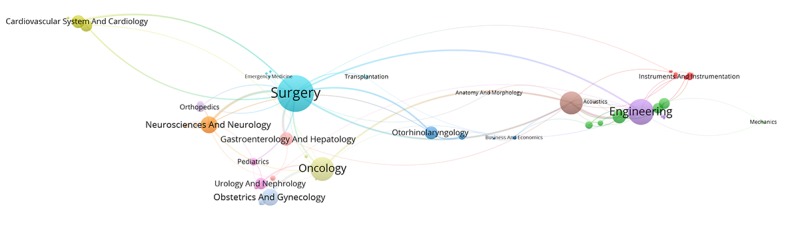
Interdisciplinary collaboration on robotics in surgery-related research from 2008 to 2012.

**Figure 5:**
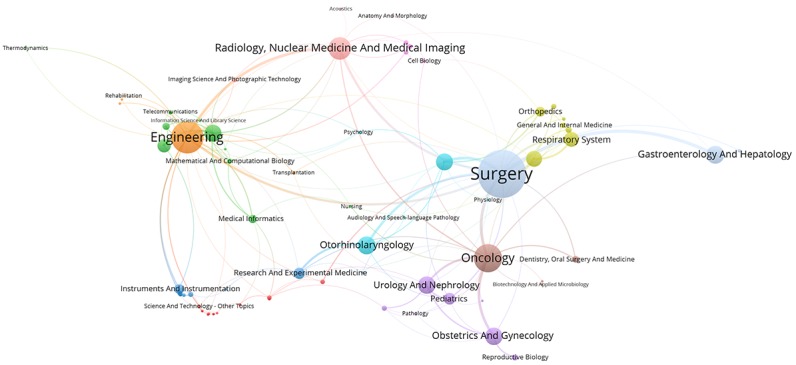
Interdisciplinary collaboration on robotics in surgery-related research from 2013 to 2017.

**Figure 6:**
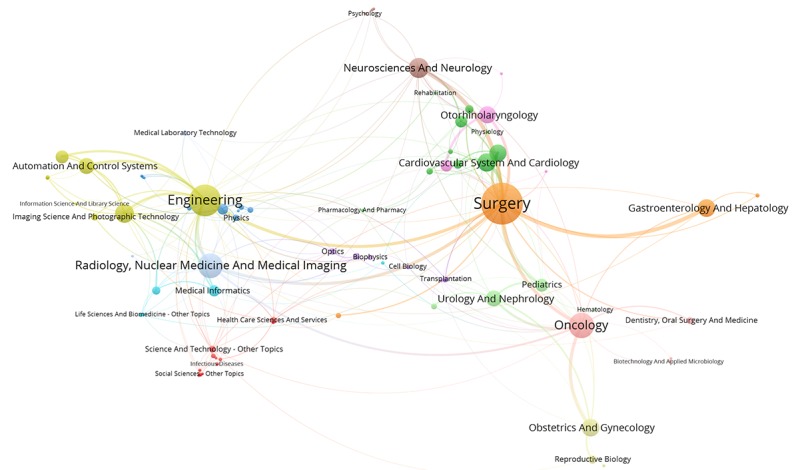
Interdisciplinary collaboration on robotics in surgery-related research from 1997 to 2017.

Figures 2 to 6 visualize the interdisciplinary collaboration for RS-related research for each period of time and overall (for detailed clusters, see Multimedia Appendix 2). The links between disciplines have been clearly shown in proportion, and the collaboration of disciplines within the cluster is significantly more than that between clusters. Although the number of clusters changed over time, there were still several clusters in each period of time, each of which has 1 or several major disciplines. For example, Surgery, Oncology, Engineering, and Urology and Nephrology appear most frequently, each of which leads a cluster in each of the maps. Thus, such a cluster is the main research direction in the field of RS. In general, the main disciplines from 1997 to 2017 were Surgery and Urology and Nephrology, with a frequency of 2802 and 1837, respectively, accounting for 45.06% (4639/10295) of the total frequency in the period of time.

**Table 2:** The overall distribution of disciplines and clusters.

Time period	Time span	Number of papers	Number of disciplines	Number of clusters	Number of discipline occurrences in papers	Mean disciplines in each paper
1997-2002	6	217	34	10	347	1.60
2003-2007	5	665	55	10	974	1.46
2008-2012	5	1985	64	15	2563	1.29
2013-2017	5	4865	85	12	6411	1.31
1997-2017	21	7732	91	13	10295	1.33

#### Network Analysis of Interdisciplinary Collaboration

**Figure 7:**
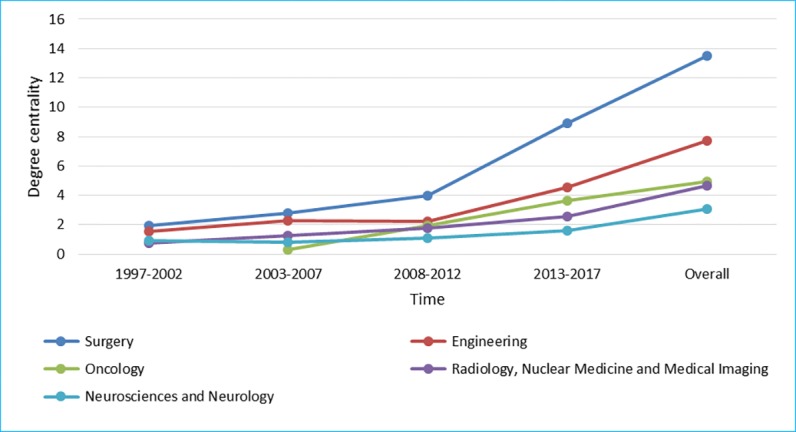
Degree centrality of top 5 disciplines.

**Figure 8:**
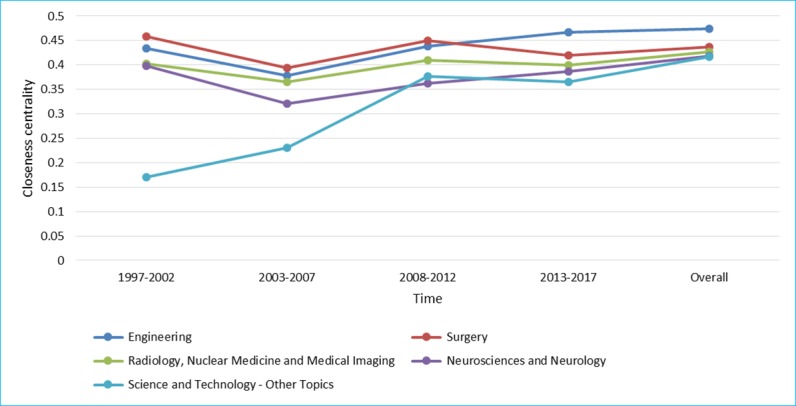
Closeness centrality of top 5 disciplines.

**Figure 9:**
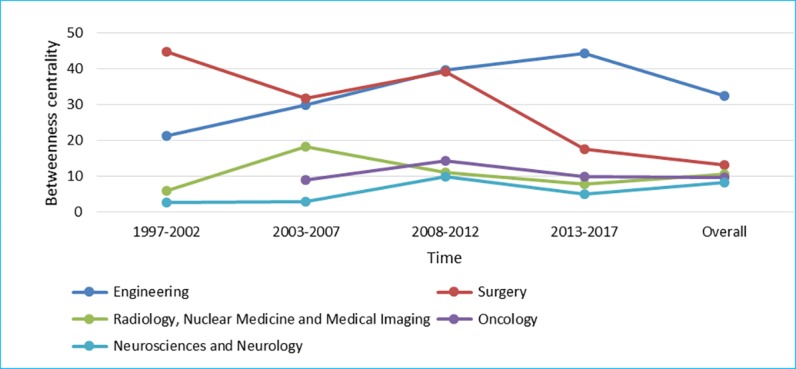
Betweenness centrality of top 5 disciplines.

Figures 7 to 9 show degree centrality, closeness centrality, and betweenness centrality for top 5 disciplines, which have held top 5 centralities over the years from 1997 to 2017 in different periods of time. The degree centrality of the top 5 disciplines is increasing continuously, whose trend is similar to the closeness centrality; although, there is a significant difference between them. The betweenness centrality of different disciplines intersected over time; there is no obviously upward or downward trend. From the perspective of degree centrality, the impact of Surgery on RS-related research is significantly higher than other disciplines. From the perspective of closeness centrality, Science and Technology played a more important role in RS-related research in the first 10 years. However, the evolution trend of the betweenness centrality is significantly different from the degree centrality and the closeness centrality, which shows that the position of top 5 disciplines, as an intermediary bridge, is not fixed.

**Figure 10:**
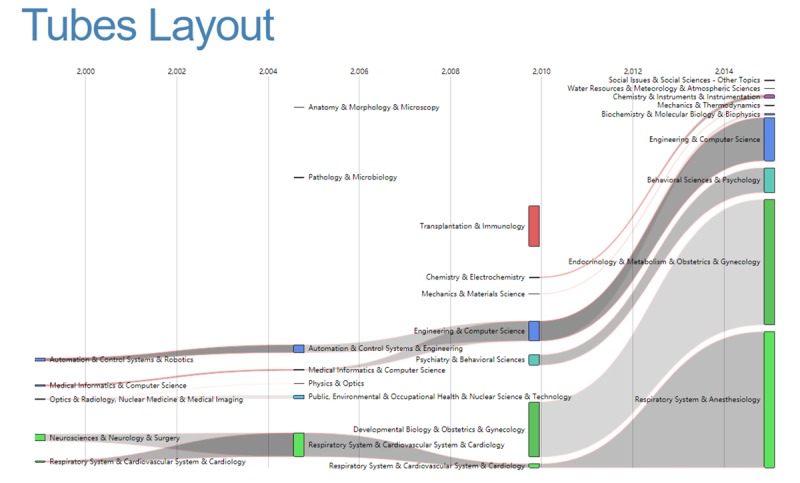
Evolution of discipline clusters over time.

### Evolution of Discipline Clusters Over Time

Figure 10 shows the overall evolution of discipline clusters related to RS over time. The 4 columns represent the 4 periods of time above. For example, the fourth column represents the data from 2013 to 2017. The cluster related to Cardiovascular System and Cardiology was integrated with other disciplines to form a larger cluster during the period from 2003 to 2007, which was independent over the years from 1997 to 2002. Optics was separated from Radiology, Nuclear Medicine, and Medical Imaging from 2003 to 2007 to form an independent cluster with Physics. Obstetrics and Gynecology and other disciplines were merged into a larger cluster during the years from 2013 to 2017. Transplantation and other related disciplines constituted a relatively independent cluster in the time period from 2008 to 2012. Respiratory System was integrated with Anesthesiology to form a larger community with Anesthesiology from 2013 to 2017. In addition, the records in Psychology and Behavior Sciences are in an increasing trend from 2008 to 2017. It should be noted that Engineering and Computer Science were merged into a cluster in the period from 2008 to 2012, then gradually grew over time. In general, the results are consistent with the above analysis to some extent.

### Research Hotspots

#### Distribution of Research Topic

There are 7 clusters for 88 high-frequency keywords in the field of RS. The name was refined according to the main keywords contained in each cluster, as shown in Table 3. Cluster 1 is mainly related to robotic-assisted laparoscopy and some applicable diseases, mainly including gynecological and bladder diseases; Cluster 2 refers to the related technologies involved in surgical robots such as microsurgery, image-guided surgery, navigation, and telesurgery; Cluster 3 focuses on the robotic-assisted laparoscopic surgery and some applicable disease, such as colorectal and gastric disease; Cluster 4 is about the da Vinci robot and transoral robotic surgery; Cluster 5 is associated with prostate diseases and corresponding surgery; Cluster 6 is related to the training of surgical robot; and Cluster 7 refers to kidney diseases and corresponding surgery.

**Figure 11:**
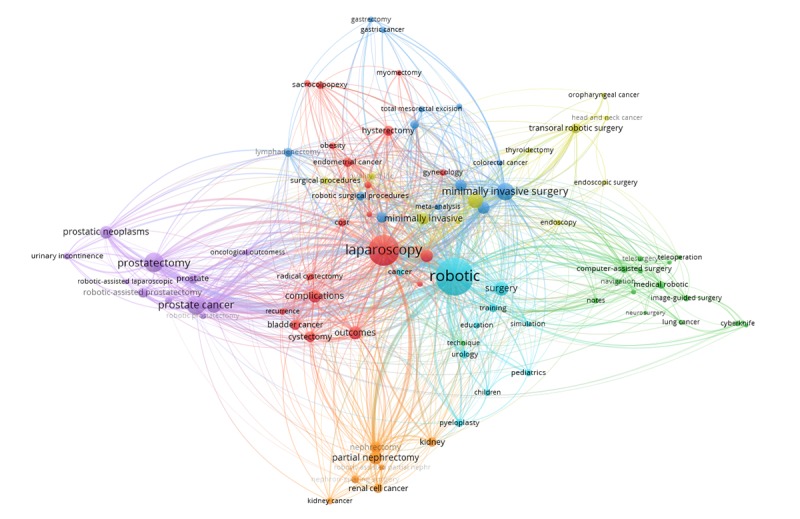
Visualization of the original 88×88 co-occurrence matrix.

**Figure 12:**
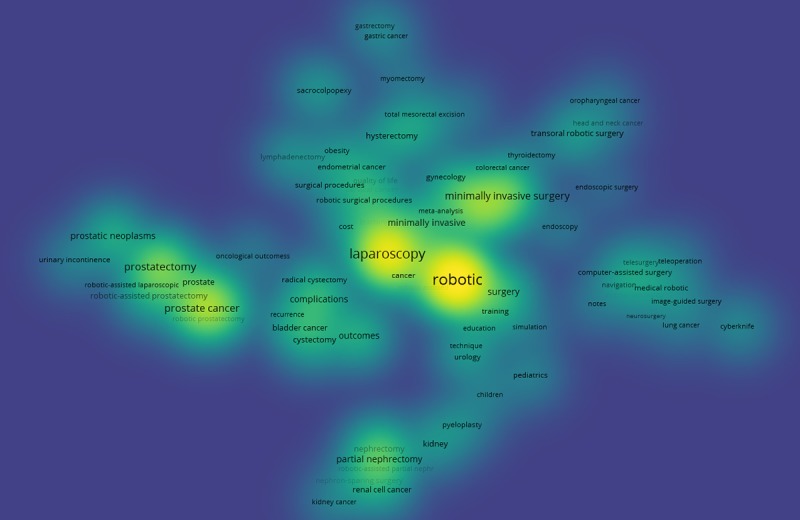
Co-occurrence density map.

All of these clusters are visualized in Figure 11. Each of the 7 colors represents a cluster. For example, the purple on the left side represents Cluster 5, of which, the keywords are mainly related to prostatectomy; the green, located in the right represents Cluster 2, which covers keywords related to techniques of computer-assisted surgery. These 7 clusters of keywords may be better identified in a density visualization (Figure 12), which immediately reveals the general structure. It can be seen that laparoscopy and minimally invasive surgery are the most important keywords in the RS-related research, in addition to robotic.

In general, the above 7 clusters of keywords on RS-related research can be combined into 3 themes according to the main content covered in each cluster: (1) various technologies, which include Cluster 2 and Cluster 6; (2) the robotic systems (ie, device and software) and related applications in surgery, which mostly contain Cluster 1, Cluster 3, and Cluster 4; and (3) prostate and kidney diseases and their corresponding operations, which include Cluster 5 and Cluster 7.

**Table 3:** 7 clusters of robotics in surgery-related research.

Cluster	Number of keywords	Cluster name	Keywords
1	19	Robotic-assisted Laparoscopy and some applicable diseases	Laparoscopy; robotic-assisted; complications; outcomes; endometrial cancer; hysterectomy; cystectomy; bladder cancer; cervical cancer; sacrocolpopexy; cost; radical cystectomy; pelvic organ prolapse; robotic-assisted laparoscopy; obesity; gynecology; myomectomy; robotic assisted; recurrence
2	16	Related technologies involved in surgical robots	Medical robotic; computer-assisted surgery; image-guided surgery; cyberknife; lung cancer; radiosurgery; navigation; telesurgery; microsurgery; augmented reality; notes; neurosurgery; haptics; teleoperation; technique; stereotactic radiosurgery
3	13	Robotic-assisted laparoscopic surgery and some applicable diseases	Minimally invasive surgery; robotic-assisted surgery; laparoscopic surgery; learning curve; rectal cancer; robotic surgical procedures; lymphadenectomy; gastric cancer; colorectal surgery; meta-analysis; colorectal cancer; gastrectomy; total mesorectal excision
4	12	da Vinci robot and transoral robotic surgery	da Vinci robot; transoral robotic surgery; minimally invasive; quality of life; endoscopy; head and neck cancer; endoscopic surgery; surgical procedures; surgical technique; oropharyngeal cancer; thyroidectomy; robotic thyroidectomy
5	11	Prostate diseases and corresponding surgery	Prostate cancer; prostatectomy; radical prostatectomy; robotic-assisted prostatectomy; prostatic neoplasms; prostate; robotic prostatectomy; robotic-assisted laparoscopic prostatectomy; urinary incontinence; oncological outcomes; continence
6	10	Training of surgical robot	Robotic; surgery; training; pyeloplasty; cancer; urology; pediatrics; simulation; children; education
7	7	Kidney diseases and corresponding surgery	Partial nephrectomy; nephrectomy; renal cell cancer; kidney; nephron-sparing surgery; robotic-assisted partial nephrectomy; kidney cancer

**Figure 13:**
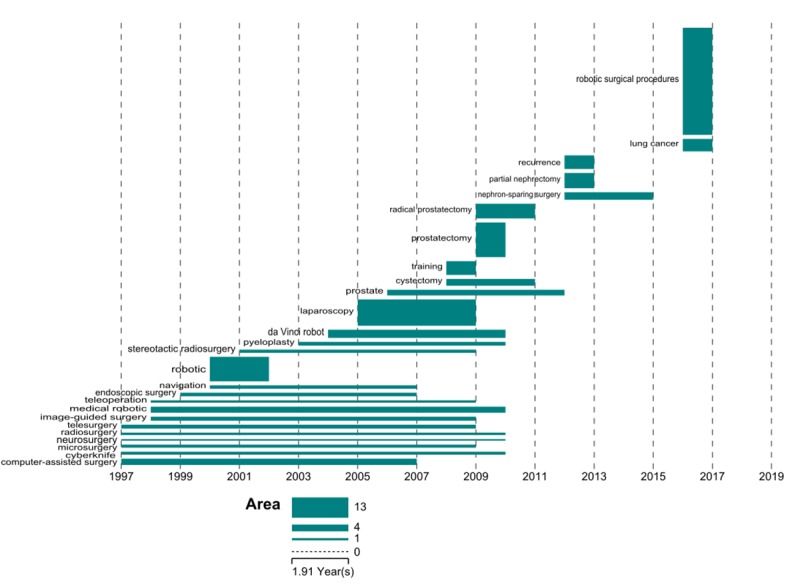
Temporal bar graph for burst keywords.

#### Temporal Bar Graph for High Frequency and High Burst Keywords

There are 26 keywords whose burst strength is more than 4 and the frequency is not less than 40 (for details, see Multimedia Appendix 3). All 26 keywords represent the frontiers of research on RS to some extent. In addition, the frequencies of these 26 keywords are 5849, showing that 0.23% (26/11,303) of keywords account for 16.02% (5849/36,505) of the total 36,505 frequencies.

The temporal bar graph of 26 keywords visually demonstrates the evolution of the topic on RS-related research over time. As shown in Figure 13, it can be seen that early research mainly focused on computer-assisted surgery, microsurgery, image-guided surgery, and medical robotic during the period from 1997 to 2007, indicating that people began to pay attention to the application of computer technology in surgery. From 2000 to 2009, the representative burst keywords were robotic, da Vinci robot, laparoscopy, and prostate, suggesting that the various robotic systems began to appear and assisted the diagnosis and surgery of some diseases. The major burst keywords from 2009 to 2015 were prostatectomy, radical prostatectomy, and partial nephrectomy, showing that the robot was mainly used in the excision of various tissues and organs. In the past 2 years, the burst keywords were robotic surgical procedures and lung cancer. It is noteworthy that the term “robotic surgical procedures” was included in Medical Subject Headings terms in 2015, suggesting that the terms of RS have been standardized, and surgical robots have been applied to a wider field of treatment of tumor and cancer.

## Discussion

### Principal Findings

This study analyzed distribution of the literature related to RS, presented that the United States plays a leading role in the publication output, and presented that the Yonsei University in South Korea published the highest number of RS-related publications. Moreover, the disciplines covered by RS have expanded rapidly over the past years, the total number of which is more than 90. There exist some core disciplines in the field of RS, all of which have extensive cooperation with other disciplines. Obviously, these 4 disciplines make the majority of contributions to RS-related research. Furthermore, there are 7 clusters for the topic hotspots related to RS with imbalanced status, the evolution of which can be divided into 3 areas.

In this study, the distribution of RS-related research is imbalanced, although the RS-related research has caused global concerns. From the perspective of the country, the G7 (the United States, the United Kingdom, Germany, Canada, Italy, France, and Japan), which are headed by the United States, occupied a dominant position in this field. The research and development of surgical robots requires a large amount of human and financial resources. Apparently, the economic foundation plays an important role in the level of research and development [38]. Therefore, countries with high economic levels are also relatively advanced in this field. From the perspective of the institution, 8 of the top 10 institutions belong to the United States, which further illustrates that the United States holds strongest research forces related to RS. Yonsei University in South Korea ranks the first with the most publication output because there are more than 10 hospitals affiliated to Yonsei Medical College [39], Severance Hospital, as one of which is the core institution for RS-related research in Asia.

This study showed that interdisciplinary collaboration of RS is widespread and has become more and more intensive in recent years. First, the number of clusters generated by visualization is gradually stable at around 13. The close connections among these disciplines aggregated into communities indicate how they support and supplement each other. However, the average number of disciplines in each paper has been reduced from 1.61 to 1.31, suggesting that the research content of each paper is more concentrated and more stable, although the disciplines of RS-related research are increasingly extensive [40]. This study on RS is mainly about how to cure a specific disease, such as various cancers, rather than a broad study of robotic surgery. Furthermore, several disciplines, such as Surgery, Oncology, Engineering, and Urology and Nephrology, are prominent in the cluster, and their collaborations with other disciplines are relatively close, showing that the main research related to RS is developed around these directions [41]. For example, Surgery has been in a dominant position for the last 21 years because it is the research topic in this study, but the relative position of Engineering disciplines that provide fundamentals and devices for RS is gradually declining, which may be due to the introduction of new disciplines with the deepening of RS-related research.

Obviously, cooperation between core disciplines needs to be further strengthened, as well as between other disciplines and core disciplines. The main disciplines in the field of RS are relatively stable, whereas other supporting disciplines are continuously changing at each period of time [42]. Surgery; Engineering; Radiology, Nuclear Medicine, and Medical Imaging; Neurosciences and Neurology have appeared in Figures 7 to 9, suggesting that these 4 disciplines are the core disciplines in the field of RS [43]. RS-related research is a relatively emerging interdisciplinary field, with a great potential impact on many areas of health care [44]. The exchange of ideas across disciplines promotes the progress of science. Medical robotics is fundamentally a team activity, involving academic researchers, clinicians, and industry. Each of these groups has unique expertise, and success comes from effective, highly interactive partnerships drawing upon this expertise [45]. Researchers with different disciplinary backgrounds have different professional knowledge, among which the cross-cooperation can promote the progress of a certain research subject. For example, in recent years, the treatment of tumor is a research hotspot in the field of RS, which needs not only doctors in Oncology but also researchers in other disciplines，such as Surgery and Engineering, who may come from different countries and institutions. Researchers with a background of different disciplines will provide different knowledge and skills to promote research on the subject of tumor treatment in the RS-related field. Therefore, more cooperation is needed in the field of RS, and the strengthening of cooperation can lead to the integration of knowledge, which means that RS is a more comprehensive research subject that includes technologies, devices, and the treatment of diseases.

Our study showed that the research focus on RS was relatively scattered and that each cluster has its own research emphasis but, in general, they can be merged into 3 main areas. Research hotspots clustering intuitively shows the relationship between 7 clusters of keywords, each of which represents a major research topic related to RS. However, some of these clusters have something in common. The clusters can be further divided into the following 3 parts: the first is the various technologies that make RS realized, such as computer-assisted surgery and image-guiding [46,47]; the second is various kinds of robotic systems and related applications in surgery, such as da Vinci robot, robotic-assisted laparoscopy, and robotic-assisted laparoscopic surgery [48-50]; the third is the application of RS in a variety of diseases and corresponding surgery, mainly tissue and organ excision, such as prostate and bladder [51].

Moreover, the research focus of several periods of time on RS reflected in the temporal bar graph is consistent with the results of the research hotspots clustering to some extent. It can be seen that the earliest research on RS is mainly about various technologies. RS is ultimately an application-driven research field. When technologies were relatively mature and measurable, the robotic systems and their related application began to emerge and became the research frontier, and surgeons began to accept and apply them in surgery. Then, the research focus began to turn to various diseases and their corresponding surgery. Apparently, it is undeniable that the robotic systems and surgery for various diseases are complementary to each other throughout the development of RS. Correspondingly, there were 3 aspects of researches in each period of time, but the focus was different. Generally, research on RS should be further promoted in these 3 areas to better strengthen the integration between surgery and robotics.

In addition, to clearly present the frontiers of RS-related research, we reduced the burst strength of keywords to 2.5 targeted at the dataset in the recent 3 years (2015 to 2017), which mainly contains lung cancer and lobectomy [52], rectal cancer and colorectal surgery [53,54], and esophageal cancer and esophagectomy [55]. It is obvious that RS-related research has begun to play an important role in the diagnosis and treatment of various specific cancers. Clearly, as the application of robots to surgery, targeted at specific diseases, has been rapidly expanded, better regulations and standards should be developed and implemented; and methods to assess safety should be adopted in future, which is conducive to exploit the full potential of robotics in medicine, especially in surgery, for the improved welfare of society everywhere [56]. In addition, other ethical concerns will emerge as robotic technologies become more intelligent with advances in cognitive software.

### Limitations

There still exist several limitations in this study. First, it is difficult to visually observe several disciplines that are most closely related because the algorithm performed in VOSviewer stipulates that the distance of disciplines in the visualization graph is closer if more collaborations between them exist. Second, the subject categorization indexed by WoSCC may be inaccurate, which may have a certain impact on the result of research and lead to some bias for the visualization of interdisciplinary collaboration. Finally, results of the topic hotspots analysis are affected by the keyword merging. This study merely merged the keyword with a frequency more than 5, that is, there are still some synonym keywords that should be merged. All of these may have some influence on the results of the topic clustering.

### Conclusions

In this study, various bibliometric measures on RS-related research were performed using the corresponding visual tool. In all, on the base of the above study, some valuable results from RS-related research were obtained, including information on interdisciplinary collaboration and research hotspots, which offer a comprehensive understanding of RS-related research. Moreover, with the development of artificial intelligence and the further widespread application of robots to surgery, it should be reasonable to believe that the literature related to RS research will continue to grow in future. In addition, as the research develops, some new application of robots in surgery will form, which will give rise to new problems, such as the degree of interdisciplinary collaboration and its effect on research productivity, all of which are the future research focus. Furthermore, the research of application will be the strength to further improve the accuracy and safety and reduce cost, although the surgery remains the core discipline of RS-related research.

## Appendix

Multimedia Appendix 1 Top 10 countries for robotics in surgery-related research.

Multimedia Appendix 2 Clusters for interdisciplinary collaboration related to robotics in surgery.

Multimedia Appendix 3 List of keywords with burst strength more than 4 and frequency not less than 40.

## Abbreviations

AGCS: average global citation score
BICOMS: Bibliographic Item Co-Occurrence Mining System
RS: robotics in surgery
Sci2: Science of Science
TGCS: total global citation score
TLCS: total local citation score
WoSCC: Web of Science Core Collection
