# Supplementary material for: Detecting the Interdisciplinary Nature and Topic Hotspots of Robotics in Surgery: Social Network Analysis and Bibliometric Study
Source: J Med Internet Res. 2019 Mar 26;21(3):e12625. doi: 10.2196/12625 (PMC6454338; doi:10.2196/12625)
Supplement: Multimedia Appendix 1 [file jmir_v21i3e12625_app1.pdf]

Top 10 countries for robotics in surgery (RS)-related research.

Table Top 10 countries.

No.	Country	Recs[a]	TLCS[b]	TGCS[c]	ALCS[d]	AGCS[e]
1	United States	3334	21989	69049	6.60	20.71
2	Germany	694	3475	12199	5.01	17.58
3	Italy	668	4395	13828	6.58	20.70
4	South Korea	549	2719	7140	4.95	13.01
5	France	520	2189	7700	4.21	14.81
6	United Kingdom	509	2630	8768	5.17	17.23
7	People’s Republic of China	451	883	3374	1.96	7.48
8	Japan	356	849	3543	2.38	9.95
9	Canada	268	991	5070	3.70	18.92
10	Belgium	193	2025	5991	10.49	31.04

[a]Recs: number of published papers.

[b]TLCS: total local citation score.

[c]TGCS: the total global citation score.

[d]ALCS: average local citation score.

[e]AGCS: average global citation score.
