# Supplementary material for: Detecting the Interdisciplinary Nature and Topic Hotspots of Robotics in Surgery: Social Network Analysis and Bibliometric Study
Source: J Med Internet Res. 2019 Mar 26;21(3):e12625. doi: 10.2196/12625 (PMC6454338; doi:10.2196/12625)
Supplement: Multimedia Appendix 2 [file jmir_v21i3e12625_app2.pdf]

Clusters for interdisciplinary collaboration related to robotics in surgery (RS).

Table Clusters of robotic surgery for interdisciplinary collaboration related to robotics in surgery (RS).

Years	Cluster	Number of Discipline	Top3 in the discipline
1997 - 2002	1	5	Engineering、Robotics、Automation & Control Systems
	2	4	Surgery、Dermatology、Emergency Medicine
	3	4	Computer Science、Medical Informatics、 Mathematical & Computational Biology
	4	4	Cardiovascular System & Cardiology、Respiratory System、Anesthesiology
	5	4	Radiology, Nuclear Medicine & Medical Imaging、Optics、Biochemistry & Molecular Biology
	6	2	Neurosciences & Neurology、Pediatrics
	7	2	Obstetrics & Gynecology、Reproductive Biology
	8	2	Orthopedics、Sport Sciences
	9	1	Medical Laboratory Technology
	10	1	Dentistry, Oral Surgery & Medicine
2003 - 2007	1	8	Pediatrics、Oncology、Transplantation
	2	6	Engineering、Automation & Control Systems、Robotics
	3	6	Computer Science、Medical Informatics、 Imaging Science & Photographic Technology
	4	6	Radiology, Nuclear Medicine & Medical Imaging、 Environmental Sciences & Ecology、 Science & Technology - Other Topics
	5	5	Surgery、Neurosciences & Neurology、 Gastroenterology & Hepatology
	6	5	Cardiovascular System & Cardiology、Respiratory System、Anesthesiology
	7	4	Orthopedics、Sport Sciences、Physiology
	8	3	Otorhinolaryngology、Research & Experimental Medicine、 Audiology & Speech-language Pathology
	9	2	Physics、Optics
	10	2	Obstetrics & Gynecology、Reproductive Biology
2008 - 2012	1	8	Instruments & Instrumentation、Physics、Optics
	2	6	Computer Science、Automation & Control Systems、Robotics
	3	6	Otorhinolaryngology、Research & Experimental Medicine、 Health Care Sciences & Services
	4	5	Cardiovascular System & Cardiology、Respiratory System、Anesthesiology
	5	4	Engineering、Biophysics、Medical Laboratory Technology
	6	4	Surgery、Emergency Medicine、Dermatology
	7	4	Neurosciences & Neurology、Psychiatry、Behavioral Sciences
	8	3	Radiology, Nuclear Medicine & Medical Imaging、Acoustics、Anatomy & Morphology
	9	3	Urology & Nephrology、Pediatrics、

			Endocrinology & Metabolism
	10	3	Gastroenterology & Hepatology、<br>Science & Technology - Other Topics、<br>Environmental Sciences & Ecology
	11	3	Mechanics、Materials Science、Mathematics
	12	3	Obstetrics & Gynecology、Reproductive Biology、Developmental Biology
	13	3	Oncology、Dentistry, Oral Surgery & Medicine、<br>Hematology
	14	2	Orthopedics、Sport Sciences
	15	2	Transplantation、Immunology
2013<br>-<br>2017	1	13	Education & Educational Research 、<br>Health Care Sciences & Services、<br>Science & Technology - Other Topics
	2	10	Computer Science、Automation & Control Systems、<br>Medical Informatics
	3	9	Research & Experimental Medicine、<br>Instruments & Instrumentation、Physics
	4	7	Cardiovascular System & Cardiology、Respiratory System、<br>Orthopedics
	5	7	Obstetrics & Gynecology、Urology & Nephrology、Pediatrics
	6	6	Otorhinolaryngology、Neurosciences & Neurology、Psychology
	7	5	Engineering、Transplantation、Medical Laboratory Technology
	8	4	Oncology、Dentistry, Oral Surgery & Medicine、<br>Biotechnology & Applied Microbiology
	9	4	Biochemistry & Molecular Biology、Optics、Biophysics
	10	4	Radiology, Nuclear Medicine & Medical Imaging、<br>Imaging Science & Photographic Technology、<br>Anatomy & Morphology
	11	3	Thermodynamics、Mechanics、Energy & Fuels
	12	3	Surgery、Gastroenterology & Hepatology、Nutrition & Dietetics
1997<br>-<br>2017	1	15	Science & Technology - Other Topics、<br>Health Care Sciences & Services、<br>Environmental Sciences & Ecology
	2	10	Cardiovascular System & Cardiology、Respiratory System、<br>Orthopedics
	3	10	Instruments & Instrumentation、Physics、Chemistry
	4	8	Engineering、Computer Science、Automation & Control Systems
	5	8	Optics、Biochemistry & Molecular Biology、Transplantation
	6	6	Medical Informatics、Mathematical & Computational Biology、Life Sciences<br>& Biomedicine - Other Topics
	7	4	Surgery、Gastroenterology & Hepatology、<br>Education & Educational Research
	8	4	Neurosciences & Neurology、Psychology、Behavioral Sciences

	9	4	Otorhinolaryngology、Research & Experimental Medicine、Dermatology
	10	4	Oncology、Dentistry, Oral Surgery & Medicine、<br>Biotechnology & Applied Microbiology
	11	4	Urology & Nephrology、Pediatrics、Endocrinology & Metabolism
	12	3	Radiology, Nuclear Medicine & Medical Imaging、<br>Medical Laboratory Technology、Acoustics
	13	3	Obstetrics & Gynecology、Reproductive Biology、<br>Developmental Biology
