# Supplementary material for: Detecting the Interdisciplinary Nature and Topic Hotspots of Robotics in Surgery: Social Network Analysis and Bibliometric Study
Source: J Med Internet Res. 2019 Mar 26;21(3):e12625. doi: 10.2196/12625 (PMC6454338; doi:10.2196/12625)
Supplement: Multimedia Appendix 3 [file jmir_v21i3e12625_app3.pdf]

# List of keywords with burst strength more than 4 and frequency not less than 40.

**Table** List of keywords with burst strength more than 4 and frequency not less than 40.

| No. | Keyword | Frequency | Burst strength | Start date-end date |
|---|---|---|---|---|
| 1 | robotic | 1931 | 16.22 | 2000-2002 |
| 2 | laparoscopy | 1083 | 34.72 | 2005-2009 |
| 3 | prostatectomy | 417 | 11.46 | 2009-2010 |
| 4 | da Vinci robot | 342 | 16.21 | 2004-2010 |
| 5 | radical prostatectomy | 240 | 9.71 | 2009-2011 |
| 6 | medical robotic | 210 | 23.85 | 1998-2010 |
| 7 | partial nephrectomy | 200 | 5.05 | 2012-2013 |
| 8 | computer-assisted surgery | 145 | 20.85 | 1997-2007 |
| 9 | robotic surgical procedures | 123 | 35.50 | 2016-2017 |
| 10 | cystectomy | 120 | 6.76 | 2008-2011 |
| 11 | prostate | 96 | 11.31 | 2006-2012 |
| 12 | image-guided surgery | 94 | 14.55 | 1998-2009 |
| 13 | cyberknife | 86 | 12.68 | 1997-2010 |
| 14 | training | 86 | 4.56 | 2008-2009 |
| 15 | nephron-sparing surgery | 74 | 7.44 | 2012-2015 |
| 16 | pyeloplasty | 71 | 8.59 | 2003-2010 |
| 17 | lung cancer | 70 | 4.13 | 2016-2017 |
| 18 | radiosurgery | 68 | 10.58 | 1997-2010 |
| 19 | navigation | 59 | 7.78 | 2000-2007 |
| 20 | telesurgery | 57 | 15.39 | 1997-2009 |
| 21 | microsurgery | 52 | 12.10 | 1997-2009 |
| 22 | endoscopic surgery | 48 | 9.46 | 1999-2007 |
| 23 | neurosurgery | 46 | 6.64 | 1997-2010 |
| 24 | teleoperation | 45 | 8.62 | 1998-2009 |
| 25 | recurrence | 45 | 4.50 | 2012-2013 |
| 26 | stereotactic radiosurgery | 41 | 8.42 | 2001-2009 |
